# Impact of a Knowledge Translation Intervention on Physical Activity and Mobility in Older Adults (the Move4Age Study): Randomized Controlled Trial

**DOI:** 10.2196/15125

**Published:** 2020-02-11

**Authors:** Sarah Neil-Sztramko, Jenna Smith-Turchyn, Julie Richardson, Maureen Dobbins

**Affiliations:** 1 School of Nursing McMaster University Hamilton, ON Canada; 2 Faculty of Kinesiology & Physical Education University of Toronto Toronto, ON Canada; 3 School of Rehabilitation Science McMaster University Hamilton, ON Canada

**Keywords:** physical activity, mobility limitation, aging, knowledge translation, randomized controlled trial

## Abstract

**Background:**

The McMaster Optimal Aging Portal (the Portal) was launched in 2014 as a knowledge translation (KT) tool to increase access to evidence-based health information.

**Objective:**

The purpose of this study was to understand if and how dissemination of mobility information through the Portal impacts physical activity (PA) in older adults.

**Methods:**

In this randomized controlled trial, participants (n=510) were assigned to a 12-week mobility-focused KT intervention or self-serve control group. The intervention included weekly email alerts and a study-specific social media hashtag linking to mobility-focused Portal materials. The control group was able to access the Portal on their own but did not receive targeted KT strategies. Participants completed questionnaires (including the Rapid Assessment of Physical Activity to quantify PA) at baseline, end of the study, and 3-month follow-up.

**Results:**

Participants were predominantly female (430/510, 84.3%), mean age 64.7 years, with no baseline differences between groups. Over half (277/510, 54.3%) of the participants were classified as “active” at baseline. There was no significant between-group difference in the PA category. Overall, both groups increased their PA with improvements maintained at 3-month follow-up (*P*<.001). In planned subgroup analyses, the KT intervention had a significant effect for those with poor or fair baseline self-rated health (*P*=.03).

**Conclusions:**

No differences were found between those who received the targeted intervention and a control group with self-serve access to the Portal, except in subgroups with low self-rated health. Both groups did report increases in PA that were sustained beyond participation in a research study. Findings suggest that different KT strategies may be needed for different types of users, with more intense interventions being most impactful for certain groups (ie, those with lower self-rated health).

**Trial Registration:**

ClinicalTrials.gov NCT02947230; https://clinicaltrials.gov/ct2/show/NCT02947230

## Introduction

Physically active lifestyles are important for healthy aging, enhancing physical mobility and independence, and reducing risk for many chronic diseases [1,2]. Despite physical activity (PA) guidelines, 94% of Canadians older than 60 years are sedentary for more than 8 hours per day [3], and more than a third of Canadians aged 65 years or older report a mobility disability [4]. Mobility disability is characterized by frequent transitions between states of mobility independence and mobility limitation (disability) [5]. This can include a decline in the frequency of performing certain activities or a modification in the way one performs certain activities, and it is often indicative of poor overall health status [6]. Although declines in indicators of mobility, such as slowing of walking speed (gait speed), is seen with normal aging, such changes predict both survival [7,8] and independence [9].

Increasingly, many people turn to the internet and social media as a source of health information [10-14]. Unfortunately, much of the Web-based health information available is not based on scientific evidence and, therefore, is unlikely to produce the intended health benefits [15,16]. Members of the public may not have the knowledge, skills, or time to sift through and identify credible messages [17-19] and, thus, may be acting on recommendations, which are unlikely to improve their health. Evidence from recent systematic reviews suggests that websites and social media have the potential to improve health behaviors, self-efficacy, and health outcomes in older adults [20], and social media interventions may positively impact health outcomes [21]. However, it is not known if access to high-quality information about maintaining and improving physical mobility results in lifestyle behavior change in older adults.

The McMaster Optimal Aging Portal (the Portal) was launched in English in 2014, and in French in 2017, as a knowledge translation (KT) tool to increase public access to trustworthy health information [22-26]. KT has been defined as “a dynamic and iterative process that includes synthesis, dissemination, exchange and ethically sound application of knowledge to improve the health of Canadians, provide more effective health services and products and strengthen the health care system” [27]. The Portal helps readers to access synthesized evidence-based resources, identify trustworthy messages, and understand scientific findings. Topics related to mobility are of interest to users: the categories “arthritis and joint conditions” and “exercise” are consistently in the monthly top 10 most-accessed lists. On the basis of the monitoring of website and email subscription analytics, users are engaging with the Portal; now we want to know if easy-to-understand, evidence-based messages change what people know and do to stay healthy and mobile.

The purpose of this study was to understand if and how the KT strategies used to disseminate information relevant to increasing PA and maintaining and improving mobility via the Portal impacts knowledge, behavioral intentions, and health among middle-aged and older Canadian adults.

## Methods

### Study Design

This 2-arm, parallel-group randomized controlled trial (RCT) was conducted to explore the effect of KT strategies for disseminating research evidence on maintaining or improving mobility to a control group who used the Portal in its existing format (self-serve control group). The study protocol was registered before study launch (NCT02947230), and no changes were made after trial registration.

### Participants

Eligible participants were adults aged 40 years or older who could read and understand English. No other eligibility criteria were applied. Participants were recruited from March to April 2017 through the Portal’s home page, weekly email alerts, and social media and online through a variety of organizations whose members are primarily middle-aged and older adults (eg, Retired Teachers of Ontario). Interested participants were directed to a study-specific website where they were given more information about the study, registered for the study, and completed the baseline questionnaire package. All procedures were reviewed and approved by the Hamilton Integrated Research Ethics Board (ID: 2444), and all participants provided informed consent.

### Study Procedures

Participants were stratified by previous Portal use and age group (<65 years or ≥65 years) and randomized in a 1:1 ratio to the KT intervention or self-serve control group. Randomization was conducted using a random numbers table in excel by a statistician not involved with any other aspects of the study. Randomization was completed after collection of all baseline data; thus, group allocation was fully concealed from both participants and study staff.

During the 12-week KT intervention, participants in the intervention group were invited to access the Portal, particularly the “Mobility and Physical Function” browse page, and received mobility-focused weekly email alerts including blog posts (short summaries of scientific evidence in a narrative format), evidence summaries (description of findings from a high-quality systematic review in lay language), and Web-resource ratings (appraisal of third-party Web-based resources) relevant to PA and physical mobility. These emails mirrored the format of the Portal’s regular weekly email subscription service, which disseminates the latest research evidence related to healthy aging to subscribers. Intervention group participants were also invited to follow a study-specific hashtag (#Move4Age) on Twitter and Facebook. Due to the publicly available nature of the Portal, control group participants were able to access the Portal in a “self-serve” fashion throughout the study period (including registering for regular Portal email alerts) but did not receive targeted KT strategies. Neither participants nor study investigators were blinded to group assignment.

### Outcome Measures

Quantitative data were collected from both groups via Web-administered questionnaires at baseline, at the end of the 12-week intervention (July 2017), and 3 months post intervention (October 2017). The primary outcome was change in self-reported PA, which was measured using the Rapid Assessment of Physical Activity (RAPA) [28]. The RAPA is a 9-item self-report scale that quantifies an individual’s level of aerobic activity into 5 categories through the RAPA1 subscale (sedentary, underactive, underactive with regular or light activities, underactive with regular activity, and active). It can also be used to classify individuals as meeting PA guidelines using the RAPA1 and RAPA2 subscales. Designed specifically for older adults, it has been shown to have similar or better sensitivity as well as positive and negative predictive value for meeting guidelines than the Behavioral Risk Factor Surveillance System PA questionnaire, and the Patient-centered Assessment and Counseling for Exercise questionnaire [28]. Secondary outcomes included level of mobility limitation, measured using the validated Manty Preclinical Mobility Disability Scale [29]; self-rated health, measured using a 5-point Likert scale, which has been found to be a reliable and valid assessment of health in the general population [30] and older adults [31]; and electronic health (eHealth) literacy, measured using the validated eHealth Literacy Scale [32]. We also assessed individuals’ knowledge of recommendations for maintaining and improving physical mobility, beliefs and attitudes toward the role of lifestyle behaviors in preventing mobility limitations, and intentions to follow published recommendations in line with the Theory of Planned Behavior [33]. Demographic data were collected including age, gender, education, diagnosis of chronic conditions, and previous use of the Portal. At the end of the study and 3 months post intervention, we collected information on participant satisfaction and use of each of the KT strategies. A qualitative process study to explore the findings from the RCT in greater depth was also conducted, with findings published elsewhere [34].

### Data Analysis

All statistical analyses were completed in SAS 9.4 (SAS Institute Inc). Baseline demographic data are summarized as mean and SD or frequency and percentage where appropriate. Independent samples *t* tests and chi-square tests were used to compare baseline characteristics between groups as well as KT strategy use and satisfaction at the end of the study and follow-up. Changes in outcome measures from baseline to the end of the study and postintervention follow-up were analyzed in an intention-to-treat fashion using a 2-way mixed effects generalized mixed model, with the interaction of intervention group by time as the main feature of interest. Participants with missing data at the end of the study or follow-up were retained in the statistical models. Subgroup analyses were planned a priori to examine potential interactions between variables of interest (previous Portal use, engagement with Portal content, and baseline self-rated health) and intervention effects, with significance set at an alpha of .05.

Using a conservative estimate of a small effect size on the RAPA (0.17, from a previous 6-week intervention conducted in older adults [35]), with a power of .80 and alpha of .05, we required a total of 388 participants in the study [36]. To account for 30% loss to follow-up, as is common in distance-based interventions, we aimed to recruit a total of 504 participants.

## Results

**Figure 1:**
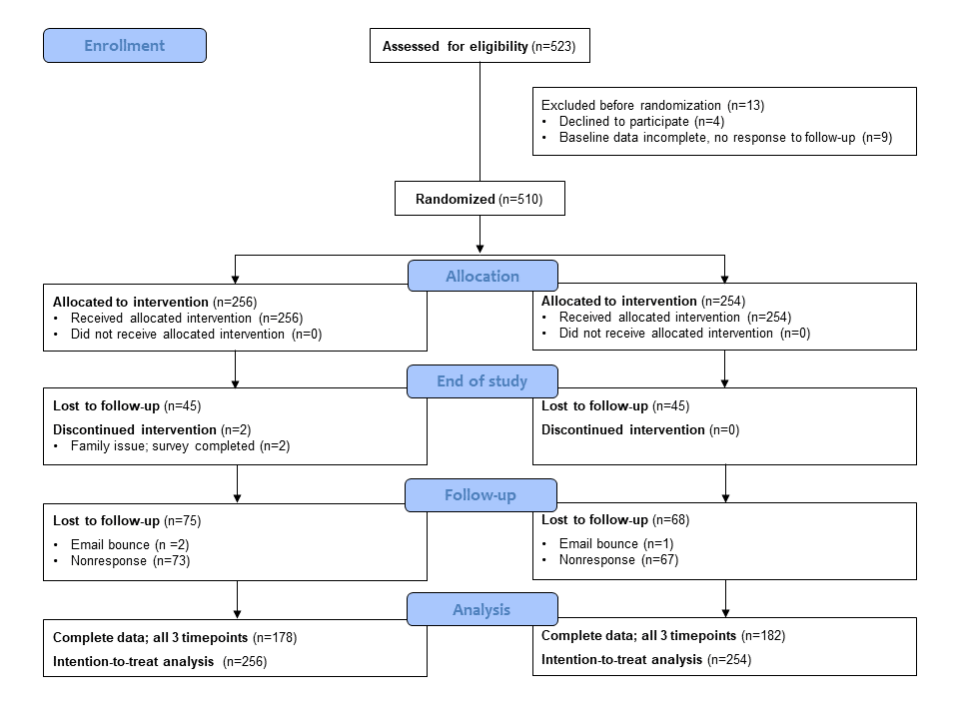
Participant flow through the study.

Participant flow through the study is displayed in Figure 1. Of the 523 individuals who responded to our call for participants, 510 provided informed consent and completed baseline questionnaires and were randomized to the intervention group (n=256) or control group (n=254). Participant characteristics are displayed in Table 1. The mean age of the participants was 64.7 years, with the majority female (430/510, 84.3%), well-educated (474/510, 92.9% had completed postsecondary education), and living in urban settings (422/510, 82.7%). There were no baseline differences between groups, with the exception of the proportion of participants who reported a fall in the last 6 months (41/256, 16.0% vs 62/254, 24.4% in the intervention vs control group; *P*=.02). There were no differences in the number of falls or the proportion of participants who visited a health care provider because of a fall.

There was no difference between the intervention and control groups in the number of participants lost to follow-up. Participants who did not complete the end-of-study (17.6%) or follow-up (31.6%) questionnaires were more likely to have never used the Portal, be employed full time, and live in rural locations than those who completed the study. There were no other differences in participant characteristics or baseline values for study outcomes between those who did and did not complete questionnaires at all 3 time points (data not shown). No adverse events were reported by participants during the study period.

**Table 1:** Participant characteristics.

Variables		Total (N=510)	Intervention (n=256)	Control (n=254)
Age, mean (SD)		64.7 (8.3)	64.7 (8.5)	64.6 (8.2)
**Gender, n (%)**				
	Male	80 (15.7)	38 (14.8)	42 (16.5)
	Female	430 (84.3)	218 (85.2)	212 (83.5)
**Education, n (%)**				
	High school diploma or less	36 (7.1)	18 (7.0)	18 (7.1)
	College diploma	111 (22.0)	58 (23.1)	53 (20.9)
	Bachelor’s degree	217 (43.1)	104 (41.4)	113 (44.7)
	Postgraduate degree	140 (27.8)	71 (28.3)	69 (27.3)
**Employment status, n (%)**				
	Retired	304 (59.7)	157 (61.6)	147 (57.9)
	Full-time employment	121 (23.8)	60 (23.5)	61 (24.0)
	Part-time employment	65 (12.8)	28 (11.0)	37 (14.6)
	Long-term disability	6 (1.2)	1 (0.4)	5 (2.0)
	Other	13 (2.6)	9 (3.5)	4 (1.6)
**Geography, n (%)**				
	Urban	422 (82.7)	209 (81.6)	213 (83.9)
	Rural	74 (14.5)	41 (16.0)	33 (13.0)
	Not reported	14 (2.7)	6 (2.3)	8 (3.1)
Self-rated health “Excellent” or “Very Good,” n (%)		303 (59.4)	144 (56.3)	159 (62.6)
Chronic disease, n (%)		283 (55.7)	141 (55.3)	142 (56.1)
**Drinks alcohol, n (%)**		414 (82.0)	211 (83.4)	203 (80.6)
	Drinks per week, mean (SD)	5.3 (5.0)	4.9 (4.3)	5.6 (5.6)
**Fall in the last 6 months, n (%)**		103 (20.2)	41 (16.0)	62 (24.4)
	Number of falls, mean (SD)	1.6 (1.2)	1.4 (0.9)	1.7 (1.3)
	Visited a health care provider because of fall, n (%)	35 (33.3)	15 (36.6)	20 (31.2)
**Previous Portal use, n (%)**				
	Never used	172 (33.8)	87 (34.0)	85 (33.6)
	Regular user	153 (30.1)	76 (29.7)	77 (30.4)
	Used occasionally	184 (36.1)	93 (36.3)	91 (36.0)
Sought information about improving mobility from a health care provider or other source in the last year, n (%)		220 (43.1)	118 (46.1)	102 (40.2)

Changes in PA are listed in Table 2. There were no significant between-group differences at the end of the study (*P*=.09) or follow-up (*P*=.07). Both groups were more likely to be categorized in a higher PA level using the RAPA at the end of the study or baseline (intervention: odds ratio [OR] 3.35, 95% CI 2.04-5.49; control: OR 1.86, 95% CI 1.14-3.03), with improvements sustained at follow-up compared to baseline (OR 3.27, 95% CI 1.96-5.47; control: OR 1.67, 95% CI 1.01-2.77). There were no between- or within-group differences in the proportion of participants classified as meeting Canada’s PA guidelines at either time point. The proportion of participants that reported they self-monitored PA was higher at the end of the study compared to baseline (intervention: OR 3.56, 95% CI 2.06-6.18; control: OR 3.05, 95% CI 1.76-5.27) and follow-up compared to baseline (intervention: OR 3.33, 95% CI 1.89-5.87; control: OR 2.04, 95% CI 1.17-3.55), but there were no differences observed between the intervention and control groups. A similar pattern was observed for the level of mobility disability using the Manty Preclinical Mobility Disability Scale (Table 2).

**Table 2:** Quantitative outcomes at baseline, end of the study, and follow-up among intervention and control participants.

Variable		Baseline		End of the study					Follow-up			
		Intervention	Control	Intervention	Control	*P* value^a^		Intervention		Control	*P* value^a^	
**Rapid Assessment of Physical Activity, % (95% CI)**							**.09**					**.07**
	Active	53.9 (47.4-60.4)	58.8 (52.8-64.8)	66.5 (61.6-71.5)	65.0 (59.9-70.2)			66.3 (61.2-71.5)		64.0 (58.5-69.6)		
	Underactive regular	25.6 (21.2-30.0)	23.6 (19.5-27.8)	21.2 (18.2-24.1)	21.6 (18.3-24.8)			21.2 (18.2-24.3)		21.9 (18.4-25.3)		
	Underactive light	15.5 (12.6-18.5)	13.5 (10.4-16.6)	9.5 (6.4-12.6)	10.3 (7.2-13.5)			9.6 (6.5-12.8)		10.9 (7.6-14.2)		
	Underactive	3.5 (2.2-4.8)	3.0 (2.0-4.0)	2.2 (1.4-3.0)	2.3 (1.5-3.2)			2.2 (1.4-3.1)		2.5 (1.6-3.3)		
	Sedentary	1.5 (0.4-2.5)	1.1 (0.2-1.9)	0.6 (0.1-1.1)	0.7 (0.1-1.3)			0.6 (0.1-1.2)		0.7 (0.1-1.4)		
Meets PA^b^ guidelines, % (95% CI)		27.4 (24.8-30.0)	28.4 (25.7-31.2)	30.8 (27.2-34.5)	32.0 (27.9-36.0)	.94		31.5 (27.4-35.5)		32.5 (28.0-36.9)	.88	
Self-monitors PA, % (95% CI)		47.4 (40.9-53.8)	54.0 (47.6-60.4)	64.3 (58.0-70.6)	68.0 (62.2-73.9)	.69		63.5 (56.9-70.1)		63.3 (56.8-69.9)	.22	
**Manty Preclinical Mobility Disability Scale, % (95% CI)**							**.59**					**.19**
	No limitation	60.2 (52.9-67.5)	53.1 (44.4-61.9)	68.0 (64.0-72.0)	65.3 (59.9-70.8)			66.5 (61.5-71.4)		65.7 (60.3-71.1)		
	Preclinical disability	7.8 (4.1-11.4)	10.7 (7.4-14.0)	3.9 (1.5-6.3)	5.2 (2.1-8.2)			4.6 (1.8-7.5)		5.0 (2.0-8.1)		
	Minor limitation	22.9 (18.5-27.3)	23.5 (17.1-29.9)	22.6 (21.0-24.3)	22.8 (20.1-25.4)			22.7 (20.5-24.9)		22.8 (20.3-25.2)		
	Major limitation	9.2 (5.2-13.1)	12.7 (7.3-18.1)	5.5 (3.4-.5)	6.7 (3.7-9.7)			6.2 (3.6-8.7)		6.5 (3.6-9.5)		
Self-rated health, mean (SD)		2.6 (0.1)	2.7 (0.1)	2.7 (0.1)	2.8 (0.1)	.82		2.8 (0.1)		2.7 (0.1)	.65	
Beliefs/attitudes, mean (SD)		13.3 (0.2)	13.6 (0.2)	13.9 (0.2)	13.6 (0.2)	.02		13.6 (0.2)		13.6 (0.2)	.22	
Intentions, mean (SD)		5.7 (0.6)	5.8 (0.6)	5.6 (0.6)	5.5 (0.6)	.04		5.6 (0.6)		5.5 (0.6)	.08	

^a^*P* value from generalized mixed model, group × time interaction at respective time points.

^b^PA: physical activity.

There was a significant between-group difference in participants’ attitudes toward mobility-related health behaviors at the end of the study (*P*=.02) but not at follow-up. Participant’s intentions to participate in mobility-related health behaviors declined slightly among participants in both groups, with a significantly greater decline in the control group (*P*=.04). There were no significant differences in intentions at follow-up. There were no significant between- or within-group differences for self-rated health or total knowledge score (data not shown).

As part of our planned subgroup analyses, a significant between-group difference was found at both the end of the study (*P*=.04) and follow-up (*P*=.02) for level of PA in participants with low self-rated health at baseline. No intervention effect was observed in participants with moderate-high self-rated health. There were no significant differences when the study sample was stratified by previous Portal use (data not shown).

At the end of the intervention period, participants in the intervention group were more likely to report that the Portal influenced their PA behaviors, and that Portal information influenced their decisions more often (3.42 vs 2.73 out of 7; Table 3). There was no difference between groups in the impact of the Portal on monitoring mobility or the proportion of participants who sought information about maintaining or improving mobility from a health care provider or other sources. The majority of participants in both groups reported receiving weekly email alerts from the Portal, with no difference between groups. Approximately one-third of the participants visited the Portal browse page, and 19.5% and 6.1% of participants reported using Facebook or Twitter to access Portal-related materials, respectively. No adverse or unintended events were reported by participants during or after the study period.

**Table 3:** Participant satisfaction and Portal use at the end of the study and follow-up.

Participant satisfaction and Portal use			Intervention	Control	*P* value
**Throughout the 12-week intervention period**					
	**Portal information influenced a decision about PA^a^, n (%)**		**140 (68.0)^b^**	**112 (54.5)^c^**	**<.01**
		How often?, mean (SD)^f^	3.43 (2.06)^b^	2.73 (1.90)^c^	<.001
	**Portal information influenced a decision about monitoring mobility, n (%)**		**108 (52.4)^b^**	**99 (48.4 )^c^**	**.46**
		How often?, mean (SD)^f^	2.91 (2.12)^b^	2.53 (1.90)^c^	.06
	Sought information about mobility from a health care provider, n (%)		55 (26.8)^b^	69 (32.9)^c^	.22
	Sought information about mobility from other sources, n (%)		47 (22.9)^b^	52 (24.9)^c^	.72
	Received weekly email alerts from the Portal, n (%)		198 (94.7)^b^	193 (89.4)^c^	.06
	Accessed the Portal via Twitter, n (%)		16 (7.7)^b^	10 (4.6)^c^	.27
	Accessed the Portal via Facebook, n (%)		41 (19.6)^b^	42 (19.4)^c^	.99
	Used the “Mobility & Physical Function” browse page, n (%)		72 (34.4)^b^	64 (29.6)^c^	.34
**3 months postintervention follow-up**					
	**Used the Portal to look for information related to mobility, n (%)**		**95 (52.5)^d^**	**94 (50.0)^e^**	**.71**
		How often?, mean (SD)^f^	3.52 (1.68)^d^	3.22 (1.69)^e^	.16
	How often did information influence a decision about PA?, mean (SD)^f^		3.89 (1.59)^d^	3.43 (1.73)^e^	*.*04
	How often did the information influence a decision about mobility?, mean (SD)^f^		3.86 (1.74)^d^	3.46 (1.79)^e^	.08
	**Used the Portal to look for information related to other topics, n (%)**		**89 (49.2)^d^**	**113 (59.9)^e^**	**<.05**
		How often?, mean (SD)^f^	3.61 (1.49)^d^	3.39 (1.46)^e^	.27
	Continued to receive weekly email alerts from the Portal, n (%)		143 (83.1)^d^	161 (87.5)^e^	.31
	Continued to access the Portal via Twitter, n (%)		14 (10.9)^d^	17 (13.1)^e^	.72
	Continued to access the Portal via Facebook, n (%)		49 (33.6)^d^	34 (23.6)^e^	.08
	Continued to use the “Mobility & Physical Function” browse page, n (%)		44 (28.6)^d^	56 (34.4)^e^	.32

^a^PA: physical activity.

^b^n=211.

^c^n=209.

^d^n=181.

^e^n=188.

^f^Numerical questions answered on a scale of 1 (not often) to 7 (very often).

In the 3 months following the intervention period, half of the participants in both groups reported using the Portal to look for mobility-related information, with no differences observed between groups. Participants in the intervention group were more likely to report that the Portal had influenced a decision about PA in the last 3 months (3.89 vs 3.43 out of 7; *P*=.04), whereas the control group was more likely to use the Portal to seek out information on other topics (59.9% vs 49.2%; *P*<.05). There were no differences between groups in the percentage of participants who continued to receive email alerts or access the Portal through Twitter, Facebook, or the browse page following completion of the study (Table 3).

## Discussion

This study is the first to evaluate the impact of dissemination of evidence-based information about mobility and PA through the Portal on PA and mobility outcomes. Participants in both the targeted KT intervention and self-serve control group reported increased PA after the 12-week intervention, with benefits maintained at 3-month follow-up; however, no significant between-group differences were observed. The lack of difference between groups is not surprising given the high degree of engagement with Portal materials reported by both groups; 89.4% of control group participants reported signing up for the Portal’s general weekly email alerts. Although engagement was lower for social media and Portal browsing, there were no significant differences between the targeted intervention group and control group. Although our KT intervention did focus specifically on topics related to PA and mobility, these topics are among the most common on the Portal itself, and it is likely that the control group was exposed to similar information during the study and poststudy period. Due to the nature of the Portal as an already existing Web-based resource, we were unable to include a true control group in our study. Thus, contamination across the control group may contribute to the lack of significant differences between study groups.

In planned subgroup analyses, we found a significant effect of the intervention in individuals who had low self-rated health at baseline. There are several potential explanations for this finding. It is possible that those with lower self-rated health benefited more from the targeted aspects of the KT intervention and specific content chosen. This suggests that certain subgroups may benefit from different or more tailored KT strategies (eg, medium of message delivery, including behavioral feedback), potentially in line with the barriers to PA that they face. This should be explored in future studies. Given that our study sample was relatively healthy and active at baseline, the small amount of change seen over time may be the result of a ceiling effect; perhaps those with low self-rated health had the greatest potential for change.

A number of behavior change theories suggest that provision of information alone is inadequate to result in long-term behavior change of a sufficient magnitude to affect long-term health outcomes [37]. On the basis of the Theory of Planned Behavior [33], attitudes toward PA and intentions to engage in activity are predictors of PA behavior. In this study, participants’ attitudes toward activity and intentions to engage in PA were significantly different between groups at the end of the study, suggesting that the targeted KT intervention had a stronger effect on these constructs. Portal materials are designed to have actionable messages within content and are specifically targeted at middle-aged and older adults. We hypothesized that this targeting would act on normative and control beliefs of participants, but further tailoring of messaging (eg, dissemination of content specific to participant characteristics or baseline knowledge or preferences) may be necessary to elicit greater behavior change. In a recent study, inner-city minority participants with type 2 diabetes were randomly assigned to an intervention delivered through a Web-based portal, which included self-management modules, health education, and social networking. Importantly, this intervention also included interaction with a telehealth nurse. At the end of the study, participants in the intervention group showed greater knowledge of diabetes and diabetes management, greater self-rated physical and mental health, greater weight loss, and improved diabetes control, although results should be interpreted with caution because of the large loss to follow-up observed in both groups [38]. These findings do, however, support our hypothesis that further tailoring and interaction with participants may increase the effectiveness of our intervention.

Although we did observe a significant within-group difference in PA throughout the study period, the absolute magnitude of the change may be considered small or moderate: an additional 12.6% of intervention group and 6.2% of control group participants were classified in the highest PA at the end of the study compared with baseline. These findings are consistent with a recent Cochrane review of computer-based weight loss or weight maintenance interventions, which found that Web-based interventions were superior to minimal intervention or control; however, they were not as effective as in-person interventions [39]. However, given the relative low cost, ease of delivery using existing Portal materials, and scalability of an intervention such as this, we believe that the small absolute change observed in this study has the potential to contribute to a meaningful difference at a population level.

An important limitation to our study is the reliance on self-report data for PA and mobility disability. Although we used a previously developed and validated tool, it is known that individuals tend to self-report higher levels of PA [40]. Due to the lack of blinding of study participants, it is possible that the intervention group had a higher degree of self-report bias; however, given the high engagement with the Portal materials in both groups, particularly around PA and mobility-related content, we believe that any overestimation of PA was similar between groups. We chose to use a self-report tool from a feasibility standpoint to be able to include a broad sample of participants across Canada. Future work could consider low-cost methods such as smartphone tracking to gather some objectively measured data.

Our study sample was relatively homogenous, consisting of relatively healthy (59.4% of participants rated their health as “Excellent” or “Very Good” at baseline), well-educated, urban-dwelling adults. Demographics of our study sample are similar to those of general Portal users previously reported by our study team [22], although our study sample was approximately 5 years younger and had a higher proportion of females. This is not surprising as approximately one-third of the study participants reported being regular Portal users at baseline, with another third reporting using the Portal occasionally. This is consistent with findings from a recent systematic review, which found that individuals with lower education as well as racial and ethnic minorities are typically less likely to use health portals [41]. More work is needed to understand how to engage these underserved groups, who may have potentially more to gain from a KT intervention such as this.

Although the Portal has been successful in engaging citizens and health care professionals, its use has not yet been evaluated with respect to changes in knowledge or behaviors. An understanding of how participants engage with both the Portal and the KT strategies is essential for ensuring the content and delivery of information through the Portal, and other health information websites will be most effective at encouraging behavior change and ultimately improving health. As highlighted by Grimshaw et al [42], the current evidence-base to guide the choice of effective KT strategies aimed at consumers to improve health outcomes is still incomplete [42]. These study findings have relevance for both individuals who use Web-based heath information resources and organizations that develop and provide it. On the basis of our findings, the KT strategies used in this study may result in improved intentions and health behaviors in particular subgroups and thus have the potential to impact a number of health outcomes, including mobility and functional independence over a longer follow-up period. More work is needed to understand which groups may benefit most from a low-cost, easily scalable intervention such as this.

## Appendix

Multimedia Appendix 1 CONSORT-EHEALTH checklist (V1.6.1).

## Abbreviations

eHealth: electronic health
KT: knowledge translation
OR: odds ratio
PA: physical activity
RAPA: Rapid Assessment of Physical Activity
RCT: randomized controlled trial
